# Quantitative and histologically validated measures of the entorhinal subfields in *ex vivo* MRI

**DOI:** 10.1093/braincomms/fcac074

**Published:** 2022-03-25

**Authors:** Jan Oltmer, Natalya Slepneva, Josue Llamas Rodriguez, Douglas N. Greve, Emily M. Williams, Ruopeng Wang, Samantha N. Champion, Melanie Lang-Orsini, Kimberly Nestor, Nídia Fernandez-Ros, Bruce Fischl, Matthew P. Frosch, Caroline Magnain, Andre J. W. van der Kouwe, Jean C. Augustinack

**Affiliations:** 1 Department of Radiology, Athinoula A. Martinos Center, Massachusetts General Hospital, Charlestown, MA, USA; 2 Harvard Medical School, Boston, MA, USA; 3 Department of Neuropathology, Massachusetts General Hospital, Boston, MA, USA; 4 CSAIL, Cambridge, MA, USA

**Keywords:** aging, Alzheimer, cytoarchitecture, segmentation, validation

## Abstract

Neuroimaging studies have routinely used hippocampal volume as a measure of Alzheimer’s disease severity, but hippocampal changes occur too late in the disease process for potential therapies to be effective. The entorhinal cortex is one of the first cortical areas affected by Alzheimer’s disease; its neurons are especially vulnerable to neurofibrillary tangles. Entorhinal atrophy also relates to the conversion from non-clinical to clinical Alzheimer’s disease. In neuroimaging, the human entorhinal cortex has so far mostly been considered in its entirety or divided into a medial and a lateral region. Cytoarchitectonic differences provide the opportunity for subfield parcellation. We investigated the entorhinal cortex on a subfield-specific level—at a critical time point of Alzheimer’s disease progression. While MRI allows multidimensional quantitative measurements, only histology provides enough accuracy to determine subfield boundaries—the pre-requisite for quantitative measurements *within* the entorhinal cortex. This study used histological data to validate ultra-high-resolution 7 Tesla *ex vivo* MRI and create entorhinal subfield parcellations in a total of 10 pre-clinical Alzheimer’s disease and normal control cases. Using *ex vivo* MRI, eight entorhinal subfields (olfactory, rostral, medial intermediate, intermediate, lateral rostral, lateral caudal, caudal, and caudal limiting) were characterized for *cortical thickness*, *volume*, and *pial surface area*. Our data indicated no influence of sex, or Braak and Braak staging on *volume*, *cortical thickness*, or *pial surface area*. The volume and pial surface area for mean whole entorhinal cortex were 1131 ± 55.72 mm^3^ and 429 ± 22.6 mm^2^ (mean ± SEM), respectively. The subfield volume percentages relative to the entire entorhinal cortex were olfactory: 18.73 ± 1.82%, rostral: 14.06 ± 0.63%, lateral rostral: 14.81 ± 1.22%, medial intermediate: 6.72 ± 0.72%, intermediate: 23.36 ± 1.85%, lateral caudal: 5.42 ± 0.33%, caudal: 10.99 ± 1.02%, and caudal limiting: 5.91 ± 0.40% (all mean ± SEM). Olfactory and intermediate subfield revealed the most extensive intra-individual variability (cross-subject variance) in *volume* and *pial surface area.* This study provides validated measures. It maps individuality and demonstrates human variability in the entorhinal cortex, providing a baseline for approaches in individualized medicine. Taken together, this study serves as a ground-truth validation study for future *in vivo* comparisons and treatments.

## Teaser

Based on ultra-high-resolution 7 T ex vivo MRI validated with extensive histological data, Jan Oltmer et. Al. parcellated the entorhinal cortex in preclinical Alzheimer's disease patients and cognitive controls and conducted subfield specific quantitative measurements in a critical time point of Alzheimer's disease progression.

## Graphical Abstract

**Graphical abstract:**
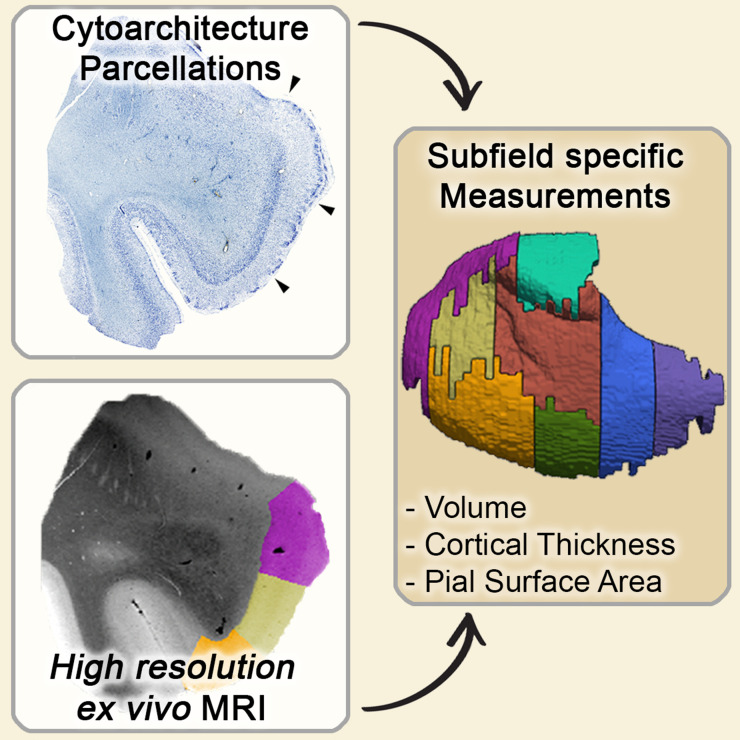

## Introduction

The human entorhinal cortex (EC) is critical for cognitive processes like spatial navigation and the encoding of time.^1,2^ Serving as a hub between the hippocampus and neocortical regions,^3^ it is indispensable for the human memory network.^4^ The EC hosts neurons particularly vulnerable to neurofibrillary inclusions. In Alzheimer’s disease, it is one of the earliest cortical areas affected with neurofibrillary tangles (misfolded and hyperphosphorylated tau protein).^5–7^ Subsequently, several stereological and neuroimaging studies have demonstrated cellular loss and atrophy in the EC before the onset of Alzheimer’s disease or in its early stages.^8–13^

In neuroimaging, the human EC has so far mostly been considered in its entirety^14,15^ or divided into a medial and a lateral region.^16^ This was supported by previous works on functional connectivity in humans, indicating distinct connectivity patterns of the medial and lateral EC.^17–19^ Yet, distinct neuroanatomical differences among EC subfields in the primate brain emphasize the subdivision into smaller subfields.^20^ Based on cytoarchitectural features, Insausti *et al.*^20^ parcellated the EC into eight subfields: olfactory subfield (EO), medial intermediate subfield (EMI), rostral subfield (ER), lateral rostral subfield (ELr), intermediate subfield (EI), caudal lateral subfield (ELc), caudal subfield (ECs), and caudal limiting subfield (ECL). With exception of the EO, the subfield functions remain unknown. Functional connectivity studies of the human EC^17,18^ rely heavily on tracer studies conducted in monkeys,^21,22^ highlighting the need for thorough parcellation based in human brain for neuroimaging application.

MRI studies provide the capability to image a whole, 3D structure with minimal distortion. Thus, *in vivo* neuroimaging established cortical thinning and loss of cortical volume as biomarkers for the diagnosis and progression of Alzheimer’s disease.^10–13^ However, compared with *ex vivo* studies, *in vivo* studies lack specificity. MRI methods provide the capability of whole-brain multidimensional measurements. Histological methods, however, provide accuracy and precision to identify subfields and their exact boundaries based on cytoarchitecture. So far, methods for the parcellation of the EC relied on voxel- or surface-based morphometry. Due to B0 distortion and signal drop-out in the temporal lobe,^23^ ground-truth measures become even more crucial.

Hippocampal volume has been routinely used as a measure of atrophy in Alzheimer’s disease. Nevertheless, hippocampal volume changes occur too late in the disease process for many potential therapies to be effective. Our study researched the EC, one of the earliest cortical areas affected by Alzheimer’s disease.^5–7^ The goal of this study was to use histologic staining coupled with *ex vivo* ultra-high-resolution 7-Tesla (7T) MRI imaging to create a comprehensive characterization of the EC subfields. It focused on tissue from cognitive controls and prodromal Alzheimer’s disease cases—a critical time point in transitioning from healthy aging to mild cognitive impairment and Alzheimer’s disease. Based on a unique dataset of ultra-high-resolution *ex vivo* MRI and validated with histologic staining, we report identification of *cortical thickness*, *volume,* and *pial surface area* of each entorhinal subfield. Subfield quantitative measures in the human EC will provide more specificity in functionality, tracking resilient healthy aging, and clinical progression of Alzheimer’s disease. Investigating how different EC subfields develop neuronal loss and atrophy in Alzheimer’s disease might not only benefit early diagnosis using *in vivo* neuroimaging biomarkers,^12,13^ but also refine the understanding of pathological progression, mechanisms, or even prevention.

## Materials and methods

### Tissue samples

Ten human brain hemispheres (five right and five left) were acquired from Massachusetts General Hospital Autopsy Suite [43–86 years; 64.75 ± 14.29 (mean ± SD); four males, four females, two unknown; post-mortem intervals <24 h]. The hemispheres were fixed by immersion in 10% formalin. All cases were immunostained for hyperphosphorylated tau and by two raters (J.C.A. and J.L.R.) evaluated for Alzheimer’s disease based on Braak and Braak (BB) staging.^5,6^ The immunohistochemistry pipeline included blocking as well as non-specific binding, primary antibody (monoclonal AT8, 1:500), biotinylated secondary antibody (goat anti-mouse, 1:200), amplification with an Avidin Biotin Complex kit, and visualization with 3′3-diaminobenzidine. Subsequently, the cases were diagnosed as four normal controls (NCs), one Braak and Braak I (BBI), four Braak and Braak II (BII), and one Braak and Braak III (BBIII). Based on clinical reports, the NC, BBI, and BBII cases were cognitively normal. The BBIII case had clinical notes of mild dementia. All cases were screened by the Massachusetts General Hospital Autopsy Suite for comorbidities^24^ and neurological, psychiatric, or infectious disease cases would have been excluded. Furthermore, all cases underwent a gross tissue inspection as well as staining (Luxol fast blue, H&E stain) to rule out vascular disease or stroke. Table 1 lists demographic information, Supplementary Table 1 lists reagents used.

**Table 1:** Demographic information of the included cases

Case #	Hemisphere	Age	Sex	PMI (h)	Braak & Braak	CERAD	MTL	Cause of death	Clinical diagnosis
							Amyloid burden		
***1***	RH	58	M	24	NC	No AD	No	Pulmonary embolism	N/A
***2***	RH	N/A	N/A	<24	NC	No AD	No	N/A	Cognitive control
***3***	RH	68	M	17	NC	No AD	No	Myocardial infarction	Cognitive Control
***4***	RH	60	M	14	BB I	No AD	Low	Aortic dissection	N/A
***5***	RH	60	M	<24	BB II	No AD	No	Liver failure	N/A
***6***	LH	43	F	24	NC	No AD	No	N/A	N/A
***7***	LH	N/A	N/A	<24	BB II	No AD	No	N/A	Cognitive control
***8***	LH	84	F	24	BB II	No AD	No	Pneumonia	Cognitive control
***9***	LH	59	F	<24	BB II	No AD	No	Lung disease	Cognitive control
***10***	LH	86	F	19	BB III	Prob. AD	Low	Cardiac arrest	Mild dementia

Cases 1–5 are right hemispheres and cases 6–10 are left hemispheres.

MTL = medial temporal lobe; PMI = post-mortem interval.

### MRI acquisition

Cases were scanned in a whole-body ultra-high-field 7T Siemens Magnetom (Siemens Healthineers, Erlangen, Germany) using two radiofrequency coil setups. Both provided similar *ex vivo* contrast and signal. The first setup was a four-turn solenoid coil (inner diameter: 28.5 mm), yielding a resolution of 100 µm isotropic.^25^ The medial temporal lobe tissue was blocked, packed into plastic Falcon tubes (50 ml, 28.5 mm diameter), and scanned in 2% paraformaldehyde solution or Fomblin. A total of seven cases were scanned using this setup. The second setup was a 7-channel phased-array receiver coil with a birdcage transmit coil yielding a resolution of 120 µm isotropic. The hemispheres were packed in a vacuum-sealed plastic bag filled with paraformaldehyde solution to reduce susceptibility artifacts. Generally, a fast-low-angle-shot (FLASH) sequence with 3D encoding (flip angles: eight cases 20°, two cases 25°) was utilized for all cases, delivering optimal contrast in post-mortem specimens to distinguish microanatomy with *ex vivo* MRI.^25^ Furthermore, three scanner runs were averaged to achieve the best possible image quality based on (i) contrast between white and gray matter, (ii) signal-to-noise ratio, and (iii) scarcity of susceptibility artifacts. The total acquisition time per case was ∼18 h. Supplementary Table 2 lists MRI parameters and coils.

### Tissue processing and histology

Histology processing was based on a previous study.^25^ First, tissue blocks were cryoprotected in 20% glycerol/2% dimethyl-sulfoxide-solution for a minimum of 10 days. The blocks were then sectioned in the coronal plane at 50 µm on a freezing sliding microtome (Leica Biosystems Inc, Buffalo Grove, IL, USA) and collected serially. A blockface photograph was captured before each section using a mounted Canon EOS-1D Mark IV camera (Canon, Tokyo, Japan) and LED ring flash. Ensuring thorough sampling, sections were sampled in series of 10 (every 500 µm), hand-mounted onto glass slides, dried overnight, and stained for Nissl substance with thionin. The staining protocol consisted of defatting (chloroform, 100% ethanol mixture, 1:1), pre-treatment (acetic acid, acetone, 100% ethanol, double distilled water mixture, 1:1:1:1), staining in buffered thionin (8%), differentiating in 70% ethanol (addition of 5–10 drops of glacial acetic acid), dehydrating in an ethanol series (70, 95, 100%), clearing in xylene, and coverslipping with Permount. Selected photomacrographs of the stained tissue were digitized using a Keyence digital microscope (Keyence Corporation of America, Itasca, IL, USA). The image quality was digitally increased by subtracting the background and adjusting the images to the optimal contrast (GIMP v2.8, The GIMP Development, https://www.gimp.org).

### Registration of MRI slices and histological sections

The manual reconciliation (matching) of MRI slices and histological sections was based on cytoarchitectural features of the EC and surrounding structures. These structures included but were not limited to the following: gyrus ambiens, collateral sulcus position/depth, hippocampal fissure position, amygdala size/shape, hippocampus size/shape, appearance of pre-subicular clouds, and dentate gyrus size/shape. Together, these landmarks help identify the individual orthogonal (i.e. coronal) levels of cut. Generally, MRI volumes were manually rotated in Freeview^26^ to match the histology and anterior–posterior spacing between MRI slices and corresponding histological sections and checked for consistency. *Ex vivo* MRI and blockface images were non-linearly registered using a fast free-form deformation algorithm (Niftyreg toolbox, University College London^27^). *Ex vivo* MRI and Nissl slides were registered manually using Freeview,^26^ taking into account translation, rotation, and scaling. See Supplementary Fig. 1 for an illustration of the registration procedures.

### Subfield parcellation

Nissl sections were manually parcellated (J.C.A. and N.S.) at approximately every 500 µm, using a Nikon SMZ1000 microscope (Micro Video Instruments Inc, Avon, MA, USA). EC as a whole corresponds to two Brodmann areas, 28 and 34. Brodmann area 34 is also known as the gyrus ambiens and Insausti’s EMI subfield. Here, we segmented the entorhinal subfields according to Insausti’s subfield protocol.^20^ Parcellated entorhinal subfields were as follows: EO, EMI, ER, ELr, EI, ELc, ECs, and ECL.^20,28,29^ See Insausti^20^ for subfield descriptions. We used the nomenclature by de Nó^30^ for layers and weighted features during evaluation. For the caudal EC subfield, we added an ‘s’ for subfield (ECs) to distinguish the subfield ECs from the whole EC. Layer II cell island characteristics were weighted most heavily, followed by Layer IV and III appearances. Furthermore, lamina dissecans width and distinctiveness, the distinctiveness of EC boundary with white matter, and anterior–posterior/medial–lateral position within EC were accounted for.

### Manual labelling and isosurface reconstruction

Based on the Nissl parcellations, EC subfields were manually labelled onto the reconciliated MRI slices using Freeview^26^ (FreeSurfer, Charlestown MA, USA). The Nissl-stained parcellations served as the ground-truth for the MRI manual labelling. Parcellated subfield labels were annotated on respective MRI slices with careful attention to not only the boundaries within the cortical ribbon, but also the pial and gray/white matter boundaries.

### Quantitative measurements

Four quantitative measurements were extracted per entorhinal subfield (EO, ER, EMI, ELr, EI, ELc, ECs, and ECL): (i) automated *cortical thickness measurements*, (ii) manual *cortical thickness measurements*, (iii) *volume*, and (iv) *pial surface area*. We describe each one in detail below.

*Automated cortical thickness measurements*: Per subfield, averaged lengths of surface normals from each vertex in the gray/white boundary mesh (created based on the isosurface) to the pial surface were measured automatically. The average EC cortical thickness was calculated by averaging thickness measurements across subfields and cases.

M*anual* c*ortical thickness measurements*: Two raters (J.O. and N.S.) measured the distance between pial surface and gray/white matter boundary. These measures were collected at three sites within each subfield and at three MRI slices per subfield (25, 50, 75% anterior/posterior extent). Measurements per subfield per case were averaged. The average EC cortical thickness was calculated by averaging thickness measurements across subfields and cases.

*Volume*: Volume equals the sum of all voxels within a given subfield MRI label multiplied by the spatial resolution (number of voxels × volume (m^3^) per voxel). The average EC volume was calculated by adding up the volume measurements of all subfields per case and averaging across cases.

*Pial surface area*: Per case, the area of the 3D-mesh pial surface model of each subfield was extracted (mm^2^). The average EC pial surface area was calculated by adding up the pial surface measurements of all subfields per case and averaging across cases.

### Statistical analysis

Statistical analysis was performed using R-Studio v.1.4.1 (The RStudio Team, https://www.r-project.org). Data were presented using Prism v.9.1 (Graphpad, https://www.graphpad.com). Multiple Shapiro–Wilk tests were computed to screen for violation of normality. An intraclass correlation (ICC; two-way random effects model, unit type average) was computed as an interrater reliability measurement between the two sets of manual cortical thickness measurements. A second ICC (same type as above) was computed as an interrater reliability measurement between the averaged manual cortical thickness measurements and automated cortical thickness measurements. Multiple Kruskal–Wallis tests were conducted to investigate differences between subfields (EO, ER, ELr, EMI, EI, ELc, ECs, and ECL), sex (male and female), and diagnosis (NC, BBI, BBII, and BBIII) in *automated cortical thickness*, *manual cortical thickness*, *volume*, and *pial surface area*. Three cases with missing data in sex were excluded from the respective analysis. In each case, Dunn’s tests were used for *post hoc* testing^31^ and corrected for multiple comparisons using the Benjamini–Hochberg Procedure.^32^ Statistical tests were two-sided and utilized an alpha level of *P* < 0.05 as the level of significance.

### Data availability

The data that support the findings of this study are available from the corresponding author upon reasonable request.

## Results

### Subfield definitions and histologic validation of *ex vivo* MRI

**Figure 1:**
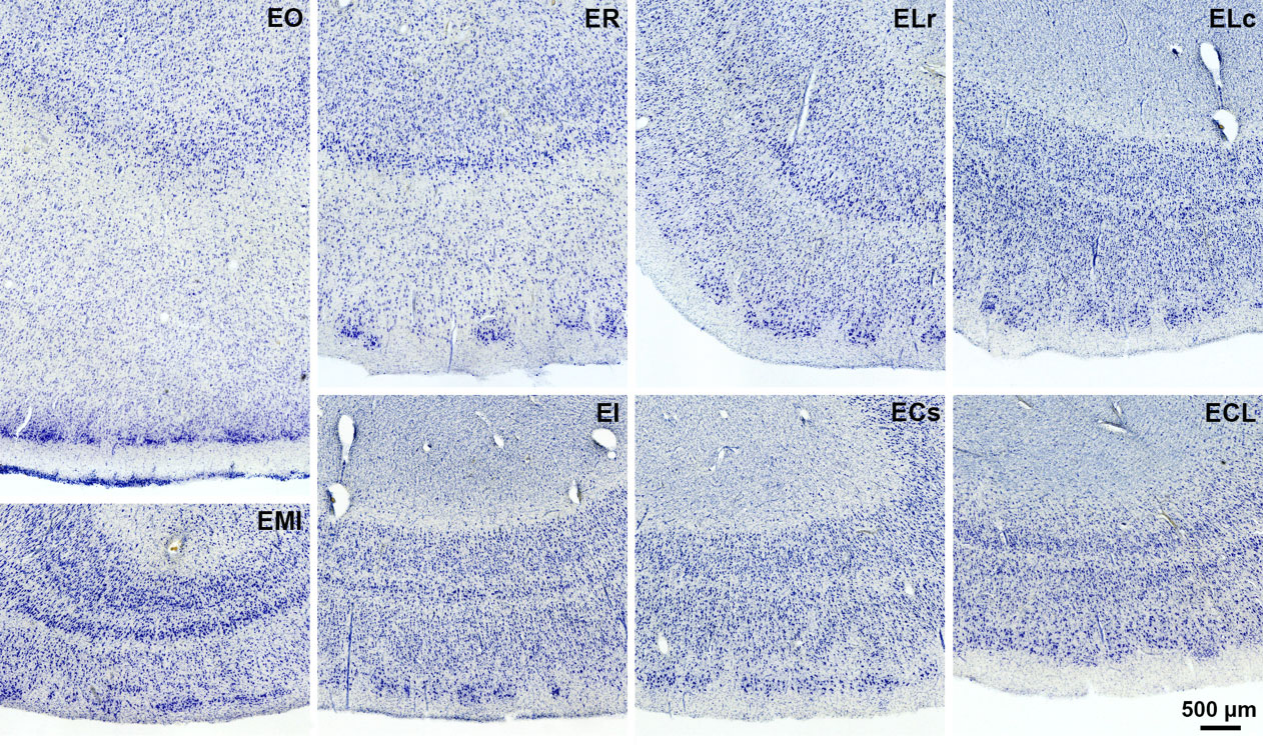
**Nissl staining**. Eight entorhinal cortex subfields displayed in coronal photomicrographs that each show distinct cytoarchitectural features. ECs = caudal subfield; ECL = caudal limiting subfield; EI = intermediate subfield; ELc = caudal lateral subfield; ELr = lateral rostral subfield; EMI = medial intermediate subfield; EO = olfactory subfield; ER = rostral subfield.

**Figure 2:**
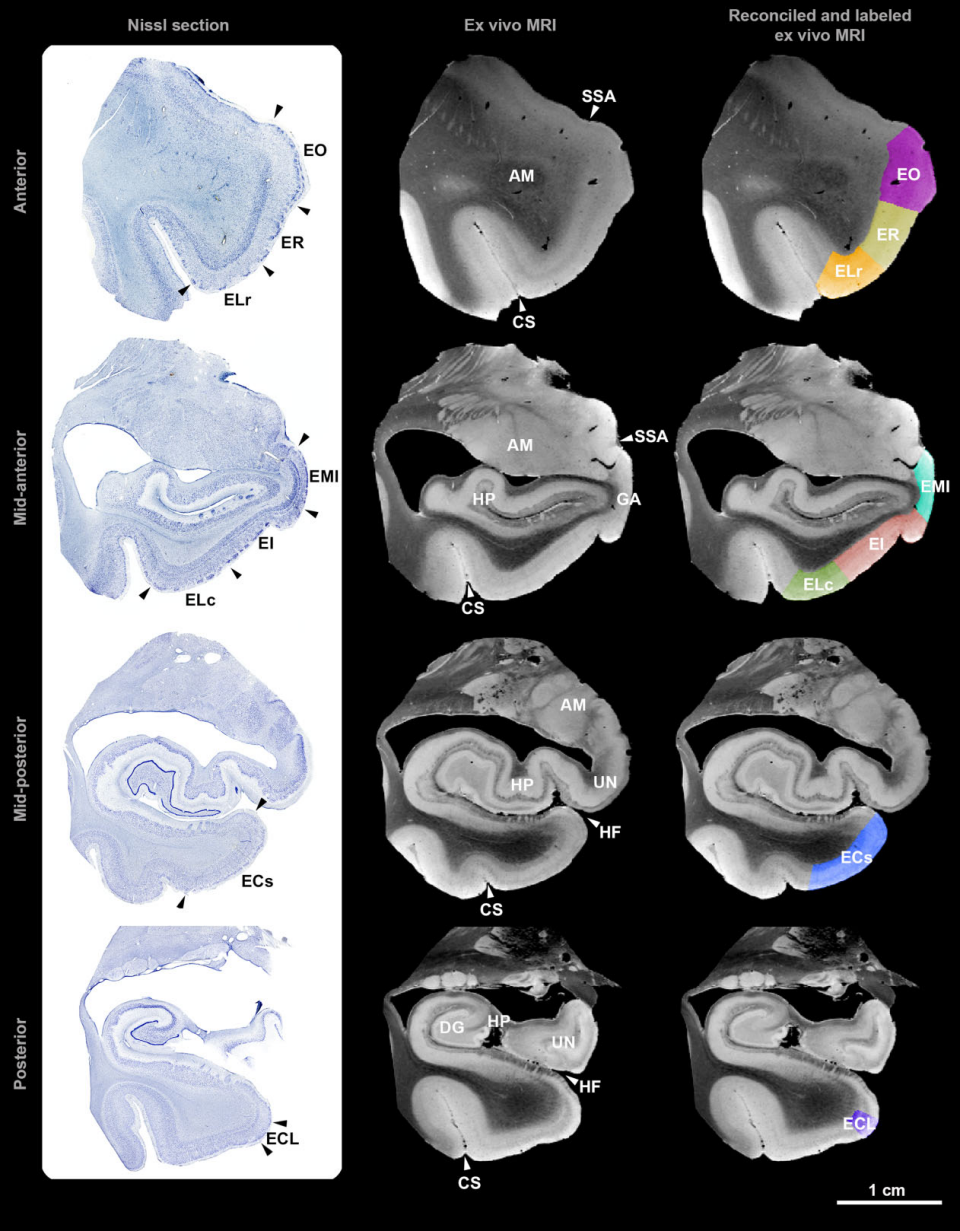
**Histologic validation of *ex vivo* MRI**. Left column: photomacrographs of Nissl-stained sections (arrowheads indicate boundaries), middle column: corresponding high-resolution *ex vivo* MRI (arrowheads indicate neuroanatomical features of the entorhinal cortex and surrounding structures), right column: reconciled and labelled *ex vivo* MRI, top row: anterior level, upper-middle row: mid-anterior level, lower-middle row: mid-posterior level, bottom row: posterior level. AM = amygdala; CS = collateral sulcus; DG = dentate gyrus; EC = entorhinal caudal; ECL = entorhinal caudal limiting; EI = entorhinal intermediate; ELc = entorhinal lateral caudal; ELr = entorhinal lateral rostral; EMI = entorhinal medial intermediate; EO = entorhinal olfactory; ER = entorhinal rostral; GA = gyrus ambiens; HF = hippocampal fissure; HP = hippocampus; SSA = sulcus semi-annularis; UN = uncus of hippocampus.

Figure 1 displays typical cytoarchitectural features of each subfield. Generally, our sample set confirmed the subfield definitions of Insausti *et al.*^20^ Yet, some minor variations were observed. We observed differences between anterior and posterior subfields in gray/white matter boundary clarity. The gray/white matter border was distinct in posterior subfields (EMI, EI, ELc, EC, and less distinct in ECL) and less distinct in more anterior subfields (EO, ER, and ELr). The latter subfields displayed a wide and diffuse gray/white matter boundary (particularly EO and ER). Similar cytoarchitectural features between ER and EO were challenging to distinguish in some cases. We observed some interindividual variability in transition zone length along the anterior–posterior axis in two cases. Case 5 showed a particularly long transition from EO to EMI and Case 10 from ER to EI. Figure 2 demonstrates correspondence and ground-truth validation between Nissl stains and *ex vivo* MRI. The Nissl-validated subfield labels show not only the boundaries within the cortical ribbon but also the pial and gray/white matter boundaries.

### Isosurface reconstructions of EC subfield labelling and interindividual variability

**Figure 3:**
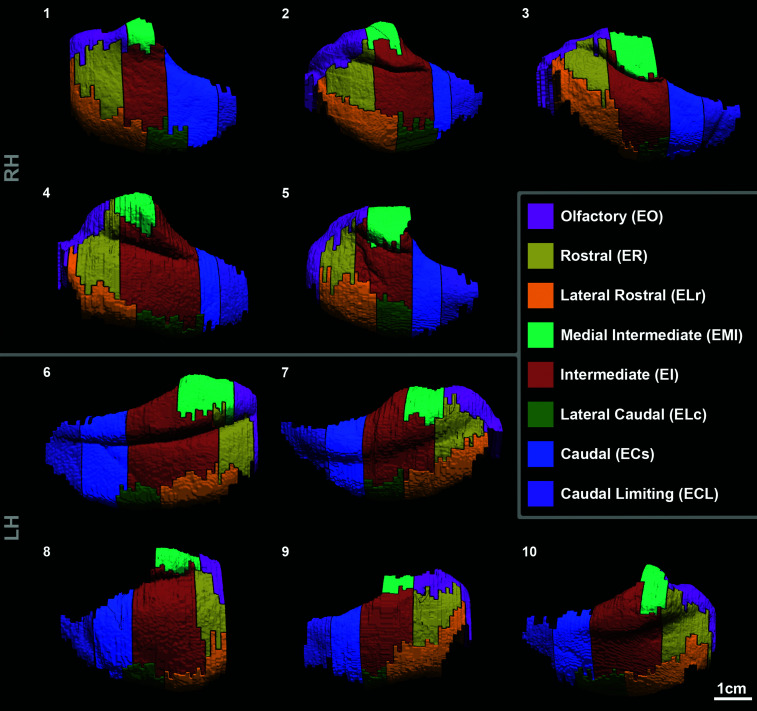
**3D isosurface reconstruction of entorhinal cortex subfield labelling**. The cases were manually labelled based on histologically validated (Nissl staining) entorhinal cortex subfields. Numbers indicate cases. Cases 1–5 are right hemispheres and cases 6–10 are left hemispheres.

Figure 3 shows all 10 isosurface reconstructions of the EC subfield labels and collectively reveals the similarities and differences among subfields in the human brain. Displaying the 3D reconstructions side by side illustrates individual variability. The overall shape of the EC varied from round to oblong (Cases 1, 2, 5, and 8 similar anterior–posterior/medial–lateral diameter; Cases 3, 6, and 7 medial–lateral less than half anterior–posterior diameter). The remaining cases fell in between (Cases 4, 9, and 10). The shape of the EC was not related to BB staging. EC subfield locations were mostly consistent across cases of different EC shapes and hemispheres. A major anatomical difference among cases was the size of gyrus ambiens (Brodmann’s area 34). It ranged from nearly absent (Case 9) to strikingly prominent (Case 4). The tentorial notch varied from shallow (Case 7) to deep (Case 2) and short (Case 2) to extending posteriorly the hippocampal fissure (Cases 2, 4, 6, and 10). An additional intrarhinal sulcus was present in three cases, located within EI (Case 5), in EI and ECs (Case 6), and within ECL (Case 3). EMI occupied the majority of gyrus ambiens and continued posteriorly past the hippocampal fissure in two cases (Cases 5 and 9). Despite this and the variable size of gyrus ambiens, we observed a low variability in size of EMI. Remarkably, ER was partially present in the gyrus ambiens in eight cases (Cases 1, 2, 3, 4, 5, 6, 7, and 10) and EO anteriorly in all cases. ELr showed some variability in how far it extended along the parahippocampal gyrus and collateral sulcus. After ECs replaced EI, ELc continued posteriorly in six cases (Cases 1, 2, 4, 6, 8, and 10). Relative to other subfields, EI and ECs show a large variability in extent from anterior to posterior. ECL was consistent in size and shape. For a video display of the 3D EC anterior-to-posterior subfields transitions in labelled coronal MRI, see Video 1.

### Quantitative measurement: subfield-specific cortical thickness

**Figure 4:**
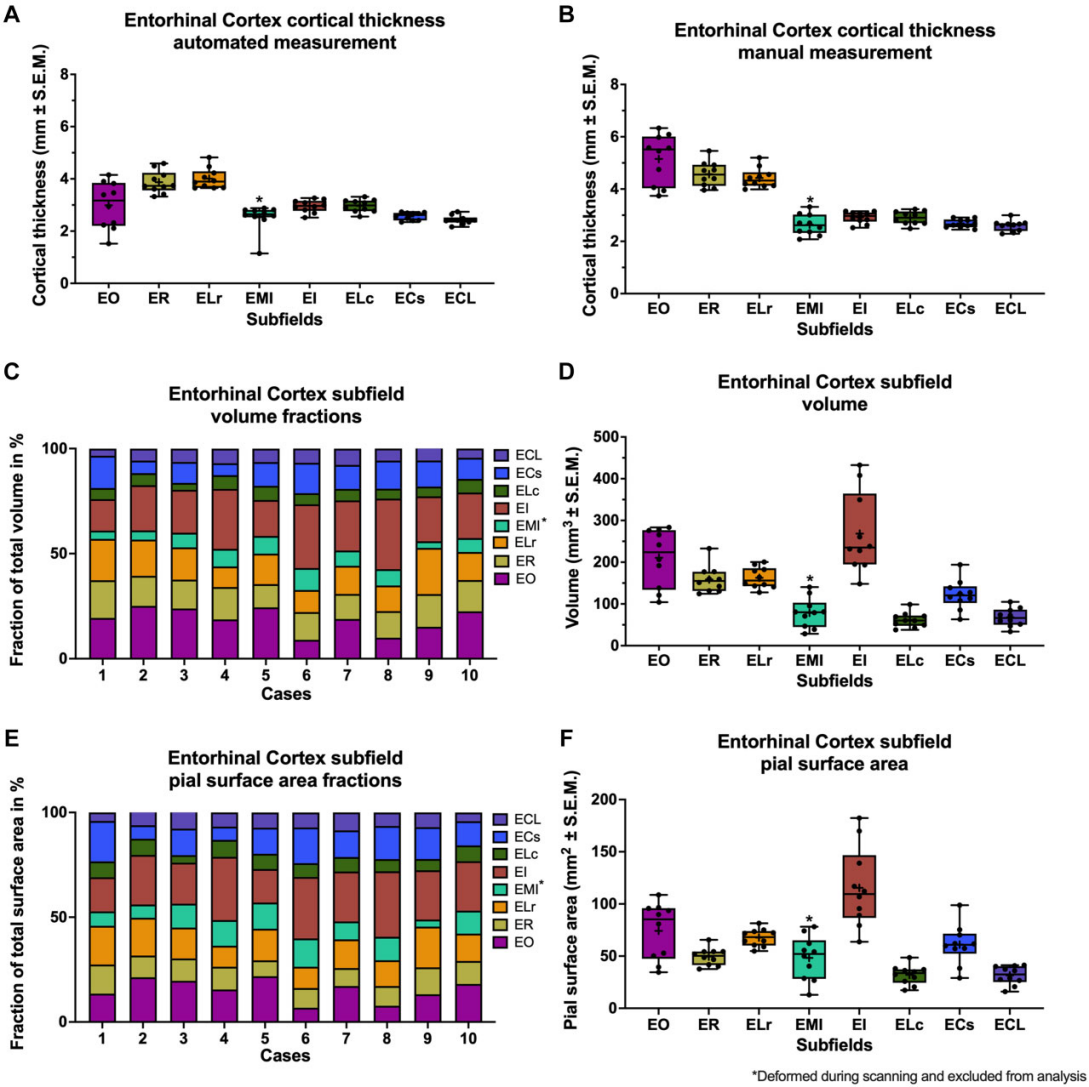
**Quantitative measurements of EC subfields**. (**A**) Automated measurements of cortical thickness of entorhinal cortex subfields. For direct comparisons, see Table 3. (**B**) Manual measurements of cortical thickness of entorhinal cortex subfields. For direct comparisons, see Table 3. (**C**) Percentages of entorhinal cortex subfield volumes relative to the whole entorhinal cortex. Cases 1–5 are right hemispheres, cases 6–10 are left hemispheres. (**D**) Volume of entorhinal cortex subfields. For direct comparisons, see Table 4. (**E**) Percentages of pial surface area of entorhinal cortex subfields relative to the whole entorhinal cortex. Cases 1–5 are right hemispheres and cases 6–10 are left hemispheres. (**F**) Pial surface area of entorhinal cortex subfields. For direct comparisons, see Table 4. Box = 25th and 75th percentile, dots = datapoints, cross = mean, line = median, whiskers = min–max.

The mean cortical thickness of the *whole EC* was 3.04±0.08 mm (mean ± SEM) in automated measurements and 3.48±0.12 mm (mean ± SEM) in manual measurements. See Table 2 for cortical thickness descriptive statistics of the EC subfields. *EO* had a mean cortical thickness of 2.99±0.28 mm, *ER* 3.87±0.14 mm, *ELr* 4.01±0.39 mm, *EMI* 2.53±0.16 mm, *EI* 2.96±0.08 mm, *ELc* 2.97±0.08 mm, *ECs* 2.57±0.05 mm, and *ECL* 2.43±0.05 mm (mean ± SEM). In manual measurements, *EO* was 5.15±0.3 mm, *ER* 4.57±0.15 mm, *ELr* 4.42±0.12 mm, *EMI* 2.64±0.13 mm, *EI* 2.92±0.07 mm, *ELc* 2.9±0.08 mm, *ECs* 2.69±0.05 mm, and *ECL* 2.6±0.07 mm (mean ± SEM). Two ICCs revealed an excellent degree of reliability between raters in manual cortical thickness measurements [ICC(A,2) = 0.99, *F*(79,79.3) = 334, *P* < 0.001] and a moderate level of agreement between automated and manual cortical thickness measurements [ICC(A,2) = 0.71, *F*(79,19.4) = 4.25, *P* < 0.001]. For correlation graphs, see Supplementary Figs 2A and B. EMI was excluded from our analysis due to deformation during scanning. We observed a main effect of subfields in *automated* and *manual cortical thickness measurements* (Fig. 4A and B) [Kruskal–Wallis H-test; automated: *χ*^2^(6) = 46.03, *P* < 0.001; manual: *χ*^2^(6) = 55.08, *P* < 0.001]. This was not the case for BB staging [Kruskal–Wallis H-test; automated: *χ*^2^(3) = 3.78, *P* = 0.287; manual: *χ*^2^(3) = 2.41, *P* = 0.49], or sex [Kruskal–Wallis H-test; automated: *χ*^2^(1) = 2.58, *P* = 0.108; manual: *χ*^2^(1) = 1.39, *P* = 0.238]. Table 3 lists direct subfield comparisons. Supplementary Table 3 lists descriptive statistics based on BB staging.

**Table 2:** Descriptive statistics of EC subfields

Variable of interest, Unit	EC Subfield	25% Percentile	Median	75% Percentile	Mean	SD	SEM	Lower	Upper
								95% CI	95% CI
Cortical thickness automated, mm	EO	2.2	3.17	3.84	2.99	0.9	0.28	2.35	3.63
	ER	3.56	3.73	4.23	3.87	0.43	0.14	3.57	4.18
	ELr	3.66	3.89	4.29	4.01	0.39	0.13	3.72	4.29
	EMI^a^	2.53	2.65	2.81	2.53	0.5	0.16	2.17	2.89
	EI	2.78	2.96	3.16	2.96	0.24	0.08	2.79	3.13
	ELc	2.76	2.98	3.17	2.97	0.24	0.08	2.8	3.14
	ECs	2.41	2.62	2.7	2.57	0.15	0.05	2.47	2.68
	ECL	2.33	2.42	2.52	2.43	0.17	0.05	2.31	2.55
Cortical thickness, manual, mm	EO	4.03	5.52	6.01	5.15	0.96	0.3	4.47	5.83
	ER	4.13	4.56	4.93	4.57	0.47	0.15	4.23	4.91
	ELr	4.13	4.33	4.64	4.42	0.38	0.12	4.15	4.69
	EMI^a^	2.32	2.62	3.02	2.64	0.4	0.13	2.35	2.92
	EI	2.76	2.97	3.11	2.92	0.22	0.07	2.76	3.08
	ELc	2.7	2.91	3.14	2.9	0.25	0.08	2.72	3.08
	ECs	2.54	2.65	2.84	2.69	0.16	0.05	2.57	2.80
	ECL	2.40	2.65	2.69	2.6	0.21	0.07	2.45	2.75
Volume, mm^3^	EO	134	224	277	211	69.2	21.9	161	260
	ER	131	155	177	159	32	1.1	136	181
	ELr	143	156	185	163	25	7.91	145	181
	EMI^a^	44.7	80	103	78.8	35.8	11.3	53.2	104
	EI	195	235	364	269	95.7	3.3	200	337
	ELc	47.7	6.3	72	61.8	17.9	5.66	49	74.6
	ECs	102	121	142	122	35.7	11.3	96.7	148
	ECL	5.3	66.5	86.2	67.9	21.7	6.87	52.3	83.4
Surface area, mm^2^	EO	47.5	85.3	95.9	74.2	27.1	8.58	54.8	93.6
	ER	41.4	5.3	54.3	49.6	8.37	2.65	43.6	55.5
	ELr	6.2	68.2	74	67.8	8.38	2.65	61.8	73.8
	EMI^a^	28.2	52.1	65.2	48.3	21.1	6.67	33.2	63.4
	EI	86.6	110	147	115	38.2	12.1	88.1	143
	ELc	24.5	33.8	37.4	32.1	9.17	2.9	25.5	38.6
	ECs	52.4	6.8	71.5	61	19.1	6.05	47.3	74.7
	ECL	25.1	32.4	4.3	31.5	8.93	2.82	25.1	37.9

Percentiles and confidence intervals for cortical thickness automated measurement, cortical thickness manual measurement, volume, and pial surface area of each EC subfield.

^a^ EMI was deformed during scanning.

**Table 3:** Cortical thickness direct EC subfield comparisons

Variable of Interest	EC	Number of datapoints	EC	Number of datapoints	Z	*P*	*P*-adjusted	*P*-adj. significance
	Subfield 1		Subfield 2					
*Cortical thickness, automated*	ECs	10	ECL	10	−0.62	0.531	0.656	Ns
	ECs	10	EI	10	1.71	0.087	0.121	Ns
	ECs	10	ELc	10	1.76	0.079	0.118	Ns
	**ECs**	10	**ELr**	10	4.57	<0.001	<0.001	****
	ECs	10	EO	10	1.59	0.111	0.146	Ns
	**ECs**	10	**ER**	10	4.3	<0.001	<0.001	****
	**ECL**	10	**EI**	10	2.34	0.019	0.034	*
	**ECL**	10	**ELc**	10	2.38	0.017	0.033	*
	**ECL**	10	**ELr**	10	5.2	<0.001	<0.001	****
	**ECL**	10	**EO**	10	2.22	0.027	0.043	*
	**ECL**	10	**ER**	10	4.92	<0.001	<0.001	****
	EI	10	ELc	10	0.04	0.965	0.965	Ns
	**EI**	10	**ELr**	10	2.86	<0.001	<0.001	*
	EI	10	EO	10	−0.12	0.904	0.949	Ns
	**EI**	10	**ER**	10	2.58	<0.001	<0.001	*
	**ELc**	10	**ELr**	10	2.81	0.005	0.015	*
	ELc	10	EO	10	−0.17	0.869	0.949	Ns
	**ELc**	10	**ER**	10	2.54	0.011	0.023	*
	**ELr**	10	**EO**	10	-2.98	0.003	0.012	*
	ELr	10	ER	10	−0.28	0.784	0.914	Ns
	**EO**	10	**ER**	10	2.70	0.007	0.018	*
*Cortical thickness, manual*	ECs	10	ECL	10	−0.42	0.668	0.739	Ns
	ECs	10	EI	10	1.18	0.240	0.336	Ns
	ECs	10	ELc	10	1.1	0.272	0.357	ns
	**ECs**	10	**ELr**	10	4.01	<0.001	<0.001	***
	**ECs**	10	**EO**	10	4.71	<0.001	<0.001	****
	**ECs**	10	**ER**	10	4.2	<0.001	<0.001	***
	ECL	10	EI	10	1.6	0.109	0.176	ns
	ECL	10	ELc	10	1.53	0.127	0.190	ns
	**ECL**	10	**ELr**	10	4.44	<0.001	<0.001	****
	**ECL**	10	**EO**	10	5.14	<0.001	<0.001	****
	**ECL**	10	**ER**	10	4.63	<0.001	<0.001	****
	EI	10	ELc	10	−0.08	0.939	0.939	Ns
	**EI**	10	**ELr**	10	2.83	0.005	0.008	**
	**EI**	10	**EO**	10	3.54	<0.001	0.001	**
	**EI**	10	**ER**	10	3.02	0.003	0.005	**
	**ELc**	10	**ELr**	10	2.91	0.004	0.007	**
	**ELc**	10	**EO**	10	3.61	<0.001	<0.001	***
	**ELc**	10	**ER**	10	3.1	0.002	0.005	**
	ELr	10	EO	10	0.70	0.482	0.595	Ns
	ELr	10	ER	10	0.19	0.852	0.894	Ns
	EO	10	ER	10	−0.53	0.606	0.706	ns

Presentation of number of datapoints, compared subfields, Z statistic, *P*-values, adjusted *P*-values, and significance for direct subfield comparisons. Computed using Dunn’s test and corrected for multiple comparisons using the Benjamini–Hochberg Procedure. Significant differences are highlighted in bold. EMI was excluded from our analysis due to deformation during scanning.

*P < 0.05, **P < 0.01, ***P < 0.001, ****P < 0.0001.

### Quantitative measurement: subfield-specific volumes

The average volume of the *whole EC* was 1131.2±55.72 mm^3^ (mean ± SEM). See Table 2 for descriptive statistics of EC subfield volumes. *EO* had an average fraction of 18.73±1.82% of the total EC volume, *ER* 14.06±0.63%, *ELr* 14.81±1.22%, *EMI* 6.72±0.72%, *EI* 23.36±1.85%, *ELc* 5.42±0.33%, *ECs* 10.99±1.02%, and *ECL* 5.91±0.40% (mean ± SEM). *EI* is prominent as the largest EC subfield. Figure 4C shows the volumetric subfield fractions for all cases. There was a significant difference in volume between subfields (Fig. 4D) [Kruskal–Wallis H-Test; *χ*^2^(6) = 52.25, *P* < 0.001]. There was no main effect of BB staging [Kruskal Wallis H-Test; *χ*^2^(6) = 1.52, *P* = 0.680], or sex [Kruskal–Wallis H-Test; *χ*^2^(1) = 0.68, *P* = 0.410]. See Table 4 for direct subfield comparisons. Supplementary Table 3 contains descriptive statistics based on BB staging.

**Table 4:** Cortical thickness direct EC subfield comparisons

Variable of interest	EC	Number of datapoints	EC	Number of datapoints	*Z*	*P*	*P*-adjusted	*P*-adj. significance
	Subfield 1		Subfield 2					
*Volume*	ECs	10	ECL	10	−1.92	0.055	0.089	ns
	ECs	10	ELc	10	−2.14	0.033	0.062	ns
	ECs	10	ELr	10	1.58	0.115	0.161	ns
	ECs	10	ER	10	1.32	0.185	0.243	ns
	**ECs**	10	**EI**	10	3.29	0.001	0.003	**
	**ECs**	10	**EO**	10	2.41	0.016	0.034	*
	ECL	10	ELc	10	−.22	0.826	0.826	ns
	**ECL**	10	**EI**	10	5.20	<0.001	<0.001	****
	**ECL**	10	**EO**	10	4.32	<0.001	<0.001	****
	**ECL**	10	**ELr**	10	3.49	<0.001	0.002	**
	**ECL**	10	**ER**	10	3.24	0.001	0.003	**
	EI	10	ELr	10	−1.71	0.088	0.131	ns
	EI	10	EO	10	−0.88	0.379	0.443	ns
	EI	10	ER	10	−1.96	0.050	0.087	ns
	**EI**	10	**ELc**	10	−5.42	<0.001	<0.001	****
	**ELc**	10	**EO**	10	4.54	<0.001	<0.001	****
	**ELc**	10	**ELr**	10	3.71	<0.001	0.001	***
	**ELc**	10	**ER**	10	3.46	0.001	0.002	**
	ELr	10	EO	10	0.83	0.407	0.450	ns
	ELr	10	ER	10	−0.25	0.800	0.826	ns
	EO	10	ER	10	−1.08	0.279	0.345	ns
*Surface area*	**ECs**	10	**ECL**	10	−2.91	0.004	0.008	**
	**ECs**	10	**EI**	10	2.48	0.013	0.027	*
	**ECs**	10	**ELc**	10	−2.98	0.003	0.008	**
	ECs	10	ELr	10	0.79	0.429	0.500	ns
	ECs	10	EO	10	0.68	0.496	0.548	ns
	ECs	10	ER	10	−0.99	0.323	0.399	ns
	**ECL**	10	**EI**	10	5.39	<0.001	<0.001	****
	ECL	10	ELc	10	−0.07	0.947	0.947	ns
	**ECL**	10	**ELr**	10	3.70	<0.001	0.001	**
	**ECL**	10	**EO**	10	3.59	<0.001	0.001	**
	ECL	10	ER	10	1.92	0.055	0.095	ns
	**EI**	10	**ELc**	10	−5.46	<0.001	<0.001	****
	EI	10	ELr	10	−1.69	0.091	0.125	ns
	EI	10	EO	10	−1.80	0.072	0.113	ns
	**EI**	10	**ER**	10	−3.47	0.001	0.002	**
	**ELc**	10	**ELr**	10	3.77	<0.001	0.001	**
	**ELc**	10	**EO**	10	3.66	<0.001	0.001	**
	ELc	10	ER	10	1.99	0.047	0.089	ns
	ELr	10	EO	10	−0.11	0.913	0.947	ns
	ELr	10	ER	10	−1.78	0.075	0.113	ns
	EO	10	ER	10	−1.67	0.095	0.125	ns

Presentation of number of datapoints, compared subfields, *Z* statistic, *P*-values, adjusted *P*-values, and significance for direct subfield comparisons. Computed using Dunn’s test and corrected for multiple comparisons using the Benjamini–Hochberg Procedure. Significant differences are highlighted in bold. EMI was excluded from our analysis due to deformation during scanning.

*P < 0.05, **P < 0.01, ***P < 0.001, ****P < 0.0001.

### Quantitative measurement: subfield-specific pial surface area

The average *whole EC* pial surface area was 479.58 mm^2^±22.6 (mean ± SEM). See Table 2 for descriptive statistics. The average fraction of *EO* on the total pial surface area was 11.8±1.21%, *ER* 15.89±1.12%, *ELr* 10.54±1.22%, *EMI* 6.43±1.01%, *EI* 29.9±1.67%, *ELc* 5.43±0.49%, *ECs* 12.42±4.03% and *ECL* 7.58±0.69% (mean ± SEM). *EI* is prominent and covers more than half of the crown of the *whole EC*. Fig. 4E illustrates pial surface fractions for each subfield. There was a main effect of subfields in pial surface area (Fig. 4F) (Kruskal Wallis H-Test; *χ2*(6) = 48.72, *P* < 0.001). We observed no main of effect BB staging (Kruskal Wallis H-Test; *χ2*(3) = 0.87, *P* = 0.832), or sex (Kruskal Wallis H-Test; *χ2*(1) = 0.35, *P* = 0.555). Direct subfield comparisons are listed in Table 4. See Supplementary Table 3 for descriptive statistics based on BB staging.

## Discussion

Neuroimaging studies have typically used hippocampal volume as the fundamental measure for Alzheimer’s disease, but hippocampal volume changes take place too late in the disease process for potential treatments. *In vivo* MRI studies have shown EC atrophy as one of the earliest volumetric changes in mild Alzheimer’s disease.^10–13^ Several parcellations of the EC have been proposed, based on different criteria and the number of subregions.^33–36^ Our work focused on the parcellation proposed by Insausti,^20^ because it was reliable, reproducible, and not overly parcellated. The goal of this study was to provide 3D measurements *within* EC at a vital tipping point in the progression of Alzheimer’s disease.^5,10,11,37^ Subsequently, our dataset consisted of cognitive controls and non-clinical Alzheimer’s disease cases. Ultra-high-resolution 7T *ex vivo* MRI neuroimaging was validated with histology-based ground-truth data, allowing us to create thorough parcellations of the human EC. This study is a logical progression from previous volumetric studies of the EC, which were primarily based on whole-brain MRI neuroimaging.^13–15,38,39^ It enables the undistorted extraction of multifaceted quantitative parameters of entorhinal subfields from 3D *ex vivo* MRI. In detail, we quantitatively measured *cortical thickness*, *volume*, and *pial surface area*—based on exact parcellations from Nissl cytoarchitecture. The application of thionin staining for Nissl substance further allowed for a more clear examination of cytoarchitectural features compared with the Kluver-Barrera stain.^40^ This resulted in potentially more exact parcellations.

We applied and confirmed the subfield definitions stated by Insausti *et al*.^20^, but observed some deviations given the thoroughness of the 3D approach. Multiple studies report the gyrus ambiens as containing only cytoarchitectonic features of EMI.^36,41^ Insausti *et al*.,^20,28^ on the other hand, identified EO and ER as being part of the rostral gyrus ambiens. Our data support this finding by Insausti *et al.*^20,28^ While EMI dominated the territory of the gyrus ambiens, EO and ER extended into it (ER: 8/10 cases, EO: 10/10 cases), indicating a more complex cytoarchitectural and functional organization. This suggests that in some humans the gyrus ambiens is formed by three subfields: EMI, EO, and ER. Insausti *et al.*^14^ reported *whole EC* mean volumes of control cases to be 1581 ± 391 and 1802 ± 323 mm^3^ (left/right hemisphere; mean ± SD). Feczko *et al*.^42^ described the EC mean *volume* of cognitively normal older adults to be 1116 ± 273 mm (connected deep collateral sulcus; mean ± SD). We observed a mean EC volume of 1131±55.72 mm^3^ (mean ± SEM), indicating lower *whole EC* mean volumes than Insausti *et al.*^14^ reported for control cases in 1998, but similar volumes as reported by Feczko *et al*.^42^ The difference in volume among studies suggests methodological differences and advances in MRI techniques in the meantime. Histology is 2D data and may present difficulties in estimating total volume. MRI provides thoroughness of quantitative measures for volume or any measure. The discrepancy between studies highlights the need for a combination of histological accuracy and ultra-high-resolution MRI methods.

Based on *in vivo* MRI, Hasan *et al*.^43^ described the mean *cortical thickness* of older cognitive controls (61–70 years) to be 3.28 ± 0.33 and 3.43 ± 0.40 mm (left/right hemisphere; mean ± SD). Similarly, Fischl *et al*.^44^ reported 3.10 ± 0.30 and 3.17 ± 0.40 mm (left/right; mean ± SEM) and Feczko *et al*.^42^ 2.76 ± 0.30 mm (connected deep collateral sulcus; mean ± SD). Our entorhinal thickness measurements were in line with this finding and revealed a mean thickness of 3.04±0.08 mm in *automated*, and 3.48±0.12 mm (mean ± SEM) in *manual cortical thickness measurements*. It is important to note that our approach was based on extensive histopathological validation of ultra-high-resolution *ex vivo* MRI. Our approach provides thorough quantitative measurements of the human EC and its subfields—not limited by spatial resolution of neuroimaging,^14,43,44^ or affected by tissue shrinkage, but with novel histological accuracy. While Insausti *et al*.^28^ based measurements on cytoarchitecture, Delgado Gonzalez *et. al*.^45^ compared MRI and histology measures, indicating associations between measurements. Our approach allows for exact quantitative measurements in 3D and is potentially more accurate than manual delineation based on lower resolution morphometry.^14^

Our data indicated significant differences in quantitative measurements between subfields. EO and EI were prominent as the most voluminous subfields (Fig. 4C; EO: 18.73±1.82%; EI: 23.36±1.85%; percentage of *whole EC* volume; mean ± SEM) and significantly more voluminous than smaller subfields such as ELc and ECL. EO and EI also covered significantly more of the pial surface area of the crown than ELc and ECL (Fig. 4E; EO: 11.8±1.21%; EI: 29.9±1.67%; percentage of entorhinal pial surface area; mean ± SEM). In *cortical thickness* that was measured manually, EO was the thickest subfield. It was significantly thicker than other subfields such as ECs and EI (Fig. 4B). This matches our qualitative observations: we observed EO to have a particularly thick cortex from pial surface to white matter and EI from medial to lateral along the cortical ribbon. We did not observe an influence of sex on quantitative measurements of the entorhinal subfields. This finding is in line with previous studies.^43,46^ Notably, the premise of this study is intended for validation findings, not multiple comparison tests. By characterizing 10 pre-clinical Alzheimer’s disease patients and normal controls, our study focused on a time point pivotal for the progression of Alzheimer’s disease. Previous stereological studies demonstrated cellular loss and atrophy in the EC before the onset or in early stages of Alzheimer’s disease.^8,9^ Yet, we did not find an influence of BB staging on *pial surface area*, *cortical thickness*^47^, or *volumetric measurements.*^10–12^ These findings provide ground-truth validation that may instigate the early detection of Alzheimer’s disease—before symptoms begin and in time for possible treatments. Future studies will have to expand these findings and apply these biomarkers to *in vivo* subjects.

The concept of individual variability of the EC has been discussed in several studies.^20,44–49^ Amunts *et al*.^48^ and Fischl *et al.*^44^ described a low degree of variability in extent and location of the EC and other reports have described more variability in the anterior EC due to variability of the rhinal sulcus.^50–52^ In our experience, most cases have a tentorial notch, but far fewer cases exhibit an intrarhinal sulcus. This was reflected in our dataset (intrarhinal sulcus: 3/10 cases). Figure 3 shows individual subfield variability from case to case and some variability in general shape. We also observed variability in transition zone length between subfields and our data indicated strong differences in variability among subfields across quantitative measurements. In general, EI and EO were prominent revealing the most extensive interindividual variability in *volume* and *pial surface area*, in contrast to ER, ELr, ELc, EC, and ECL, which displayed a small variability (Fig. 4D and F). EO displayed a large variability in *cortical thickness*. We hypothesize that this was due to individuality and long and grading gray/white matter boundaries, which has been explicitly described for EO^20,53^ (Fig. 4A and B). EI however showed a small variability in cortical thickness among cases. Differences in variability between subfields highlight the importance of multifaceted quantitative measurements in describing characteristics and differences in entorhinal subfields and in human variability.^54^

This study has some limitations. The scanning procedure yielded optimal contrast, but in some cases resulted in a compression of the gyrus ambiens due to the plastic container. Therefore, EMI was removed from formal analysis and only reported in descriptive measurements. EMI volume was not likely affected since it was compressed medial/laterally, but compensated and elongated superior/inferiorly. The delineation of ER was a second limitation due to similarity to EO and subtle transitions in some cases. The cerebral cortex transitions in a 3D fashion, which can be challenging to reproduce and view on a 2D histologic section. Even though regimented parcellation protocols and quality assessment were implemented, error margins exist. A larger sample size may lead to more fine-tuned results, especially taking into account the observed interindividual variability of the human EC. Due to methodological reasons (errant rays at the tissue edge), automated *cortical thickness* measurements tended to be more difficult in regions located on the edge, which resulted in differing results. This was especially the case for EO. Notably, automated *cortical thickness* measurements were sampled on 3D data, which generally leads to an underestimation of distances.^55^ We suspect that these together explain the difference between automated and manual *cortical thickness* measurements. Even so, manual and automated *cortical thickness* measurements were significantly correlated.

By combining the two domains of ultra-high-resolution *ex vivo* MRI and histological methods, our study provides a novel specificity for entorhinal subfield parcellation. Not limited by neuroimaging resolutions, but with histologic precision, we described and compared entorhinal *cortical thickness*, *volume,* and *pial surface area* on a subfield-specific level (Fig. 4, Table 2). The strength in our findings is not to make new revelations about sex differences, or diagnostic interpretations. We provide a cytoarchitectonic validation of quantitative measurements on the substructure level of the human EC. Our data highlights pattern, variability, and similarity among individuals in a region critical for Alzheimer’s disease. Our ground-truth approach translates histopathology into *ex vivo* MRI and serves as a validation study for future *in vivo* comparisons utilizing higher resolutions than in current standards.^56^ We created an exact parcellation *of* the entorhinal substructure, laying the groundwork for a probabilistic atlas and integration into FreeSurfer.^26^ This future work will utilize the latest neuroimaging modelling techniques. Our study provides a valuable descriptive pipeline,^54^ which in the future might increase the sensitivity for Alzheimer’s disease diagnosis based on quantitative measurements *within* EC^13,57^ and may provide a basis for individualized medicine.

## Supplementary Material

fcac074_Supplementary_Data

## Abbreviations

EC =: entorhinal cortex
ECs =: caudal subfield
ECL =: caudal limiting subfield
EI =: intermediate subfield
ELc =: caudal lateral subfield
ELr =: lateral rostral subfield
EMI =: medial intermediate subfield
EO =: olfactory subfield
ER =: rostral subfield
MTL =: medial temporal lobe
