# Supplementary material for: Quantitative and histologically validated measures of the entorhinal subfields in ex vivo MRI
Source: Brain Commun. 2022 Mar 25;4(3):fcac074. doi: 10.1093/braincomms/fcac074 (PMC9128374; doi:10.1093/braincomms/fcac074)
Supplement: fcac074_Supplementary_Data [file fcac074_Supplementary_Data.docx]

**Quantitative and Histologically Validated Measures of the Entorhinal Subfields in Ex Vivo MRI**

**Supplementary Material**

Oltmer, J*^1,2^, Slepneva, N*^1^, Llamas Rodriguez, J^1^, Greve, DN^1,2^, Williams, EM^1^, Wang, R^1^, Champion, SN^3^, Lang-Orsini, M^3^, Nestor, K^1^, Fernandez-Ros^1^, N, Fischl, B^1,2,4^, Frosch, MP^3^, Magnain, C^1,2^, van der Kouwe, AJW^1,2^, Augustinack, JC^1,2^

*These authors contributed equally to this work

**Author affiliations:**

1 Athinoula A. Martinos Center, Massachusetts General Hospital, Department of Radiology, Charlestown, MA, USA

2 Harvard Medical School, Boston, MA, USA

3 Massachusetts General Hospital, Department of Neuropathology, Boston, MA, USA

4 CSAIL, Cambridge, MA, USA

| *Reagent* | *Source* | *Identifier* |
| --- | --- | --- |
| Acetic Acid, Glacial, Certified ACS | Fisher Scientific | A38-500 |
| Acetone | Fisher Scientific | HC3001GAL |
| Chloroform, Certified ACS | Fisher Scientific | C298-1 |
| Dimethyl Sulfoxide, Certified ACS | Fisher Scientific | D128-500 |
| 100% Denatured Ethyl Alcohol | Fisher Scientific | HC8001GAL |
| 95% Denatured Ethyl Alcohol | Fisher Scientific | HC11001GL |
| Glycerol | SIGMA | G9012-1L |
| L-Lysine Hydrochloride | Fisher Scientific | BP386-100 |
| Sodium-meta-periodate 99% | Fisher Scientific | AC19838-1000 |
| Sodium phosphate dibasic dihydrate | SIGMA | 71505-250G |
| Sodium phosphate monobasic heptahydrate | SIGMA | S2429-250G |
| Paraformaldehyde 96% | Fisher Scientific | AC41678-5000 |
| Permount Mounting Medium | Fisher Scientific | SP15-500 |
| Thionin Acetate, pure, high purity biological stain | Fisher Scientific | AC22984-0050 |
| Sodium Acetate Trihydrate, ACS Reagent Grade | Fisher Scientific | RDCS0250500 |
| Xylenes, Histological Grade | Fisher Scientific | X3P-1GAL |
| Acetic Acid, Glacial, Certified ACS | Fisher Scientific | A38-500 |
| Acetone | Fisher Scientific | HC3001GAL |
| Chloroform, Certified ACS | Fisher Scientific | C298-1 |
| Dimethyl Sulfoxide, Certified ACS | Fisher Scientific | D128-500 |
| AT8 monoclonal antibody | Fisher Scientific | MN1020 |
| Peroxidase AffiniPure Goat Anti-Mouse IgG (H+L) | Jackson Immuno Research | 115-035-003 |
| 3'3-Diaminobenzidine | Vector Laboratories | SK-4100 |
| Vectasian Elite ABC kit (standard) | Vector Laboratories | PK-6100 |

**Supplementary Table 1. Reagents and sourcing:** Reagents used, source and identifier.

| *Case #* | *Magnetic*  *field*  *strength* | *Region* | *Coil* | *Scanning*  *solution* | *Voxel*  *size* | *TR (msec)* | *TE*  *(msec)* | *Flip angle*  *average* | *Averaged scans* |
| --- | --- | --- | --- | --- | --- | --- | --- | --- | --- |
| *1* | 7 T | MTL | 4-turn solenoid | Fomblin | 100 µm | 45 | 10.75 | 25 | 4 |
| *2* | 7 T | MTL | 4-turn solenoid | Fomblin | 100 µm | 45 | 10.75 | 25 | 4 |
| *3* | 7 T | MTL | 4-turn solenoid | Fomblin | 100 µm | 54 | 25 | 20 | 4 |
| *4* | 7 T | MTL | 7-channel receive coil and transmit birdcage | PLP | 120 µm | 60 | 30 | 20 | 6 |
| *5* | 7 T | MTL | 4-turn solenoid | Fomblin | 100 µm | 54 | 25 | 20 | 4 |
| *6* | 7 T | MTL | 7-channel receive coil and transmit birdcage | PLP | 120 µm | 60 | 30 | 20 | 6 |
| *7* | 7 T | MTL | 4-turn solenoid | PLP | 100 µm | 54 | 25 | 20 | 3 |
| *8* | 7 T | MTL | 7-channel receive coil and transmit birdcage | PLP | 120 µm | 60 | 30 | 20 | 3 |
| *9* | 7 T | MTL | 4-turn solenoid | PLP | 100 µm | 54 | 16.2 | 20 | 3 |
| *10* | 7 T | MTL | 4-turn solenoid | Fomblin | 100 µm | 54 | 25 | 20 | 5 |

**Supplementary Table 2. MRI acquisition parameters:** MTL: medial temporal lobe, TR: repetition time, TE: echo time.

| **Variable of interest, Unit**  **BB - stage** | **EC Subfield** | **25% Perc.** | **Median** | **75% Perc.** | **Mean** | **Std. Dev.** | **S.E.M.** | **Lower**  **95% CI** | **Upper**  **95% CI** |
| --- | --- | --- | --- | --- | --- | --- | --- | --- | --- |
| Cortical Thickness,  Automated, mm  **NC** | EO | 1.67 | 2.75 | 3.96 | 2.79 | 1.2 | 0.60 | 0.89 | 4.7 |
|  | ER | 3.42 | 3.73 | 4.31 | 3.82 | 0.49 | 0.25 | 3.04 | 4.6 |
|  | ELr | 3.77 | 4.04 | 4.41 | 4.07 | 0.34 | 0.17 | 3.54 | 4.61 |
|  | EMI* | 2.48 | 2.7 | 2.86 | 2.68 | 0.2 | 0.1 | 2.36 | 3 |
|  | EI | 2.72 | 3.02 | 3.26 | 3 | 0.29 | 0.15 | 2.53 | 3.46 |
|  | ELc | 2.76 | 2.83 | 3.09 | 2.89 | 0.19 | 0.09 | 2.6 | 3.19 |
|  | ECs | 2.41 | 2.45 | 2.64 | 2.5 | 0.13 | 0.07 | 2.29 | 2.71 |
|  | ECL | 2.3 | 2.39 | 2.47 | 2.38 | 0.09 | 0.04 | 2.24 | 2.52 |
| Cortical Thickness, Manual,  mm  **NC** | EO | 4.44 | 5.78 | 6.24 | 5.49 | 1 | 0.5 | 3.9 | 7.08 |
|  | ER | 4.48 | 4.72 | 4.87 | 4.69 | 0.22 | 0.11 | 4.35 | 5.03 |
|  | ELr | 4.44 | 4.52 | 5.04 | 4.67 | 0.36 | 0.18 | 4.1 | 5.24 |
|  | EMI* | 2.37 | 2.55 | 2.7 | 2.54 | 0.19 | 0.09 | 2.24 | 2.84 |
|  | EI | 2.67 | 2.93 | 3.13 | 2.91 | 0.24 | 0.12 | 2.52 | 3.29 |
|  | ELc | 2.69 | 2.71 | 2.96 | 2.79 | 0.17 | 0.09 | 2.51 | 3.06 |
|  | ECs | 2.54 | 2.57 | 2.83 | 2.65 | 0.18 | 0.09 | 2.37 | 2.93 |
|  | ECL | 2.32 | 2.53 | 2.68 | 2.51 | 0.19 | 0.09 | 2.21 | 2.8 |
| Volume, mm^3  **NC** | EO | 139 | 229 | 275 | 214 | 73 | 36.5 | 98.1 | 330 |
|  | ER | 154 | 169 | 177 | 166 | 12.9 | 6.43 | 146 | 187 |
|  | ELr | 150 | 181 | 192 | 174 | 23.2 | 11.6 | 137 | 211 |
|  | EMI* | 40.8 | 63.8 | 125 | 76.7 | 46.2 | 23.1 | 3.21 | 150 |
|  | EI | 168 | 233 | 366 | 255 | 109 | 54.7 | 81.4 | 430 |
|  | ELc | 41.7 | 56.5 | 68.3 | 55.5 | 13.8 | 6.92 | 33.5 | 77.5 |
|  | ECs | 76.5 | 134 | 183 | 131 | 55.3 | 27.7 | 43.1 | 219 |
|  | ECL | 40.5 | 67.2 | 86.5 | 64.7 | 24 | 12 | 26.5 | 103 |
| Surface area, mm^2  **NC** | EO | 42.8 | 74.3 | 96.3 | 71.1 | 29.3 | 14.7 | 24.5 | 118 |
|  | ER | 48.1 | 53.1 | 54.4 | 51.9 | 3.55 | 1.77 | 46.2 | 57.5 |
|  | ELr | 62.2 | 72.1 | 79.2 | 71.2 | 9.25 | 4.62 | 56.4 | 85.9 |
|  | EMI* | 27.3 | 42.1 | 72.5 | 47.3 | 24.4 | 12.2 | 8.46 | 86.1 |
|  | EI | 71.7 | 101 | 154 | 109 | 44.5 | 22.2 | 38.2 | 180 |
|  | ELc | 20.4 | 32.4 | 37.3 | 30 | 9.14 | 4.57 | 15.5 | 44.6 |
|  | ECs | 37.2 | 68.5 | 93 | 66.2 | 29.2 | 14.6 | 19.8 | 113 |
|  | ECL | 18.9 | 32.5 | 40.3 | 30.6 | 11.3 | 5.67 | 12.5 | 48.6 |
| Cortical Thickness,  Automated, mm  **BBI, BBII, BBIII** | EO | 2.3 | 3.21 | 3.84 | 3.12 | 0.73 | 0.3 | 2.35 | 3.89 |
|  | ER | 3.56 | 3.84 | 4.26 | 3.91 | 0.43 | 0.17 | 3.46 | 4.35 |
|  | ELr | 3.65 | 3.79 | 4.25 | 3.96 | 0.45 | 0.19 | 3.49 | 4.44 |
|  | EMI* | 2.21 | 2.65 | 2.79 | 2.43 | 0.64 | 0.26 | 1.76 | 3.1 |
|  | EI | 2.83 | 2.96 | 3.1 | 2.93 | 0.22 | 0.09 | 2.7 | 3.16 |
|  | ELc | 2.77 | 3.1 | 3.22 | 3.02 | 0.27 | 0.11 | 2.73 | 3.31 |
|  | ECs | 2.51 | 2.68 | 2.72 | 2.62 | 0.15 | 0.06 | 2.46 | 2.77 |
|  | ECL | 2.34 | 2.42 | 2.67 | 2.46 | 0.20 | 0.08 | 2.25 | 2.68 |
| Cortical Thickness, Manual,  mm  **BBI, BBII, BBIII** | EO | 3.89 | 5.11 | 5.7 | 4.93 | 0.95 | 0.38 | 3.93 | 5.92 |
|  | ER | 4.01 | 4.27 | 5.09 | 4.49 | 0.60 | 0.24 | 3.86 | 5.12 |
|  | ELr | 4.09 | 4.17 | 4.39 | 4.26 | 0.32 | 0.13 | 3.92 | 4.59 |
|  | EMI* | 2.18 | 2.77 | 3.13 | 2.7 | 0.50 | 0.20 | 2.17 | 3.23 |
|  | EI | 2.75 | 2.98 | 3.11 | 2.93 | 0.23 | 0.10 | 2.68 | 3.17 |
|  | ELc | 2.72 | 3.07 | 3.19 | 2.97 | 0.28 | 0.12 | 2.68 | 3.27 |
|  | ECs | 2.58 | 2.74 | 2.84 | 2.71 | 0.17 | 0.07 | 2.54 | 2.89 |
|  | ECL | 2.53 | 2.66 | 2.78 | 2.66 | 0.21 | 0.09 | 2.43 | 2.88 |
| Volume, mm^3  **BBI, BBII, BBIII** | EO | 130 | 224 | 278 | 208 | 73.6 | 30 | 131 | 285 |
|  | ER | 129 | 137 | 178 | 153 | 40.8 | 16.6 | 111 | 196 |
|  | ELr | 139 | 148 | 173 | 155 | 25 | 10.2 | 129 | 181 |
|  | EMI* | 60.3 | 80 | 103 | 80.2 | 32 | 13.1 | 46.6 | 114 |
|  | EI | 195 | 246 | 370 | 277 | 95.3 | 38.9 | 177 | 377 |
|  | ELc | 47.7 | 64.7 | 81.3 | 65.9 | 20.2 | 8.26 | 44.7 | 87.2 |
|  | ECs | 102 | 119 | 130 | 116 | 18.6 | 7.6 | 96.8 | 136 |
|  | ECL | 50.3 | 65.9 | 89.8 | 70 | 22.1 | 9.03 | 46.8 | 93.2 |
| Surface area, mm^2  **BBI, BBII, BBIII** | EO | 46.3 | 85.3 | 97.4 | 76.3 | 28.2 | 11.5 | 46.7 | 106 |
|  | ER | 39.5 | 45.2 | 57.1 | 48 | 10.6 | 4.31 | 37 | 59.1 |
|  | ELr | 59.1 | 64.5 | 74 | 65.5 | 7.73 | 3.15 | 57.4 | 73.6 |
|  | EMI* | 33.5 | 52.1 | 65.2 | 49 | 21 | 8.58 | 26.9 | 71 |
|  | EI | 86.6 | 114 | 150 | 120 | 37.2 | 15.2 | 80.7 | 159 |
|  | ELc | 24.5 | 34.3 | 40 | 33.4 | 9.77 | 3.99 | 23.2 | 43.7 |
|  | ECs | 52.4 | 58.6 | 64.1 | 57.5 | 10.6 | 4.31 | 46.4 | 68.6 |
|  | ECL | 25.1 | 32.4 | 40.3 | 32.2 | 8.07 | 3.3 | 23.7 | 40.6 |

*Deformed during scanning

**Supplementary Table 3. Descriptive statistics of EC subfields, split by Normal Control and Braak and Braak stage (BB):** Percentiles and confidence intervals for cortical thickness automated measurement, cortical thickness manual measurement, volume and pial surface area of each EC subfield, split by Normal Control (NC) and Braak and Braak staging (any Braak and Braak stage). * EMI was deformed during scanning.

**Supplementary Figure 1. Registration of blockface image, ex vivo MRI and Nissl section:** Exemplary display of registered blockface image, high resolution *ex vivo* MRI and Nissl section on different levels of the EC.

**Supplementary Figure 2. Intraclass correlation of manual and manual as well as automated and manual cortical thickness measurements: A)** Excellent reliability between both raters in manual cortical thickness measurements. **B)** Moderate agreement between automated cortical thickness measurements and averaged manual cortical thickness measurements.
